# Combined creatine and HMB co-supplementation improves functional strength independent of muscle mass in physically active older adults: a randomized crossover trial

**DOI:** 10.1007/s11357-025-01889-y

**Published:** 2025-10-10

**Authors:** Rafael Ramos-Hernández, Álvaro Miguel-Ortega, María Martínez-Ferrán, Diego Fernández-Lázaro, Natalia Busto, Juan Mielgo-Ayuso

**Affiliations:** 1 Faculty of Health Sciences, University of Burgos (UBU), 09001 Burgos, Spain; 2 Advanced Research in Integrative Physiology for Life (IAFIV) Research Group, University of Burgos (UBU), 09001 Burgos, Spain; 3 Faculty of Education, Alfonso X El Sabio University (UAX), 28691 Madrid, Spain; 4 Research Group “Nutrition and Physical Activity”, Spanish Nutrition Society “SEÑ”, 28010 Madrid, Spain; 5 Area of Histology, Faculty of Health Sciences, University of Valladolid, 42004 Soria, Spain; 6 Neurobiology Research Group, Faculty of Medicine, University of Valladolid, 47005 Valladolid, Spain

**Keywords:** Creatine monohydrate, β-Hydroxy-β-methylbutyrate, Functional strength, Sarcopenia, Older adults, Multicomponent exercise, Muscle quality, Neuromuscular adaptations, Healthy aging, Body composition

## Abstract

Sarcopenia is a major contributor to frailty and functional decline among older adults. Combining exercise with nutritional strategies such as creatine monohydrate (CRE) and β-hydroxy-β-methylbutyrate (HMB) supplementation may help to preserve strength and independence. To evaluate the effects of 6-week CRE + HMB supplementation combined with an integral physical conditioning (IPC) program on functional strength and body composition in physically active older adults. In a randomized, double-blind, placebo-controlled crossover trial, 30 older adults (20 men, 10 women; ≥ 60 years) completed two 6-week intervention periods (CRE + HMB or placebo) separated by a 3-week washout. The IPC program performed in both conditions consisted of four supervised weekly sessions combining strength, power, multicomponent circuits, high-intensity interval and moderate intensity continuous training), performed at 40–100% training heart rate (THR) and 20–90% one-repetition maximum (1RM) and structured as warm-up, main part and cooldown. Functional strength and body composition (bioelectrical impedance analysis) were assessed pre- and post-intervention, respectively. Significant time × group interactions were observed for fat mass, fat-free mass, total muscle mass, skeletal muscle mass, appendicular skeletal muscle mass, muscle mass index, skeletal muscle index and ALM/BMI (all *p* < 0.05). The CRE + HMB group showed reductions in fat mass and body fat percentage, with slight numerical increases in muscle parameters, whereas the placebo group exhibited opposite trends. However, within-group changes were not statistically significant. In contrast, CRE + HMB significantly improved multiple functional strength outcomes, including leg/back strength, arm flexion strength, upper-body endurance (dumbbell flexion, push-ups, isometric hold) and core endurance (crunches). Regression analyses suggested that these improvements were largely independent of changes in muscle mass, supporting a potential neuromuscular mechanism. Six weeks of CRE + HMB supplementation combined with IPC enhanced functional strength and endurance in active older adults, largely independent of changes in muscle mass. This combined approach represents a promising strategy for preserving functional capacity and promoting healthy ageing.

**Supplementary Information:**

The online version contains supplementary material available at 10.1007/s11357-025-01889-y.

## Introduction

Progressive loss of skeletal muscle mass and strength, known as sarcopenia, is a major contributor to frailty, functional decline and reduced quality of life in older adults [1]. This condition increases the risk of falls, dependence and mortality, representing a critical public health challenge for the aging global population [2]. Therefore, maintaining muscle strength and functional capacity is essential for promoting autonomy and healthy aging [3].

Exercise training is widely recognized as the most effective intervention to combat sarcopenia because it stimulates muscle protein synthesis and improves neuromuscular function [4, 5]. However, owing to age-related anabolic resistance, the adaptive response to exercise is often attenuated in older adults [6], highlighting the importance of combining training with additional strategies, such as nutritional support. Integral Physical Conditioning (IPC), which combines multicomponent training, functional training, strength, endurance, power, speed, coordination, balance, mobility and flexibility exercises, has gained increasing relevance in older populations. IPC targets muscular strength and improves global functional capacity, contributing to a greater reduction in fall risk and improvement in activities of daily living [7].

Creatine monohydrate (CRE) supplementation has consistently been shown to increase muscle mass, strength and functional performance in both young and older adults [8, 9]. Mechanistically, creatine not only increases intramuscular phosphocreatine content, facilitates rapid ATP synthesis and enables higher training volumes and intensities [10] but also promotes muscle protein synthesis through the activation of anabolic signaling pathways such as mTOR. Additionally, creatine may reduce muscle protein breakdown, induce cell volumization by increasing intracellular water content, and potentially attenuate oxidative stress and inflammation, thereby contributing to improved muscle recovery and function [11, 12].

β-Hydroxy-β-methylbutyrate (HMB), a metabolite of leucine, exhibits potent anti-catabolic properties by downregulating the ubiquitin–proteasome proteolytic pathway and supporting muscle preservation [13, 14]. HMB has also been shown to activate anabolic signaling via mTOR and improve muscle cell membrane integrity, contributing to reductions in muscle damage marker levels [15]. While HMB alone generally produces modest effects on lean mass gains in older adults, evidence suggests that it helps preserve muscle mass and strength, mitigate sarcopenia progression and support functional capacity during periods of high catabolic stress or disuse [14, 15].

The complementary mechanisms of action of CRE and HMB provide a strong rationale for investigating their combined supplementation, especially in older adults, where anabolic resistance and muscle loss pose significant challenges to maintaining functional independence and quality of life. Recent studies in elite athletes have reported additive effects of CRE + HMB supplementation on strength, body composition and muscle damage markers [16–18]. Fernández-Landa et al. (2020) demonstrated that this combination improved athletic performance, attenuated exercise-induced muscle damage and favorably modulated anabolic and catabolic hormonal responses in elite endurance athletes [17, 18]. However, to date, there is a lack of studies evaluating these combined supplementation effects in older adults, despite promising evidence in athletes.

Furthermore, while increases in muscle mass are traditionally assumed to drive strength gains, recent evidence suggests that improvements in functional performance can also result from neuromuscular adaptations, independent of hypertrophy [19]. Understanding whether strength improvements are primarily mediated by muscle mass changes or functional adaptations is critical for developing optimized intervention strategies for older adults.

Despite promising evidence in athletes [16–18], two critical gaps remain. First, no studies have evaluated CRE + HMB co-supplementation in physically active older adults, a population in which potential synergistic effects counteract age-related anabolic resistance. Second, the specific contribution of neuromuscular adaptations versus hypertrophy to strength gain with this combination remains unexplored, although neural adaptations play a pivotal role in strength improvement in older adults [20].

This study addresses these gaps by investigating (i) the efficacy of 6-week CRE + HMB supplementation combined with Integral Physical Conditioning (IPC) on functional strength in physically active older adults, and (ii) whether observed strength improvements are primarily mediated by changes in muscle mass or neuromuscular adaptations. We hypothesized that CRE + HMB supplementation, alongside IPC, will enhance functional performance mainly through nonhypertrophic mechanisms, providing a novel strategy to support independence and mitigate functional decline in aging.

## Materials and methods

### Study design and participants

This study was designed as a randomized, double-blind, placebo-controlled crossover trial involving 30 physically active older adults (62.68 ± 5.28 years; range: 60–82 years; 20 men and 10 women). An a priori power analysis was conducted in G*Power (v3.1.9.7) using an ANOVA repeated-measures, within–between interaction test (two groups, two measurements: PRE and POST; correlation among repeated measures = 0.50; nonsphericity correction = 1). Assuming a medium effect size (*f* = 0.25), *α* = 0.05 and 1–*β* = 0.80, the required total sample size was *N* = 54 for a parallel-group design. The crossover design, in which each participant acted as their own control, substantially reduced inter-individual variability and provided comparable power with fewer participants. Forty participants were initially recruited to account for attrition, and the final analyzed sample (*n* = 30) achieved an observed power of 78.4% for the primary analyses, with effect sizes (*η*^2^*p* = 0.17–0.42) exceeding the a priori assumption (*f* = 0.25), and attrition limited to 10%, below the 15–20% typically reported in aging research. This methodology aligns with established guidelines for efficient crossover trial designs [21].

A family of co-primary endpoints was pre-specified, comprising changes in functional strength (handgrip strength, leg/back dynamometry, arm flexion and muscular endurance tests) and body composition parameters (fat mass, fat-free mass and sarcopenia-related indices). In line with the revised European Working Group on Sarcopenia in Older People (EWGSOP2) and other international guidelines [1], low muscle strength is considered the primary indicator of sarcopenia, with grip strength (kg) widely recommended as the most reliable and clinically representative measure for diagnosis, while low appendicular skeletal muscle mass (ALM) or ALM adjusted for BMI is used to confirm the condition. Accordingly, the a priori sample size calculation was based on grip strength as a representative endpoint, while acknowledging that other indices such as ALM or ALM/BMI are also part of the operational definition. The choice of a representative endpoint reflects the fact that correlations among outcomes mitigate the effective penalty on power compared with assuming independence. Secondary endpoints included exploratory analyses of neuromuscular adaptations using regression models and sex-specific effects.

**Fig. 1:**
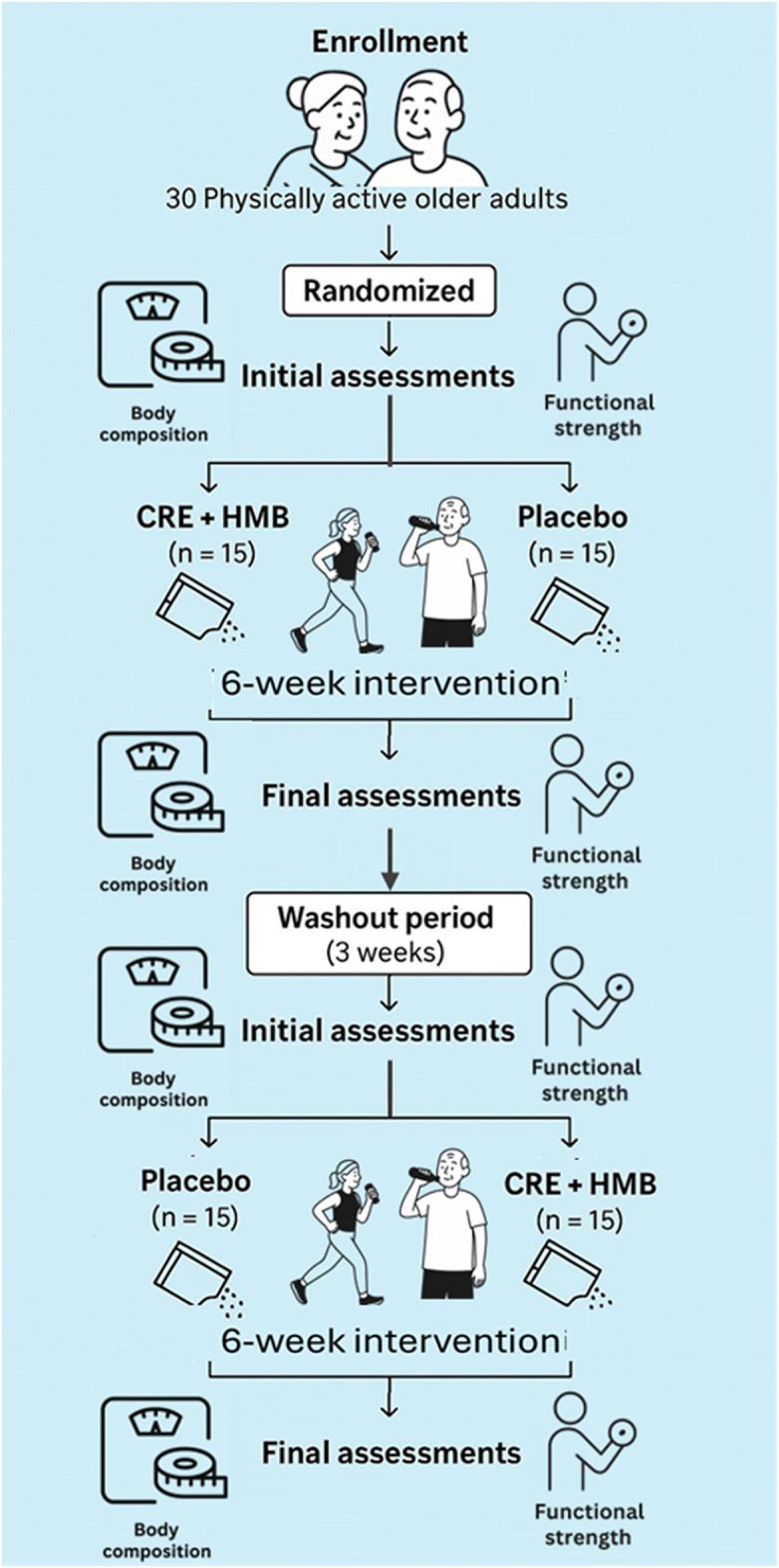
The study design flowchart illustrates the crossover protocol, including initial anthropometry and strength assessments, 6 weeks of exercise with either CRE plus HMB supplementation or placebo, a 3-week washout period and subsequent crossover to the alternate condition

Participants were randomized into two groups (*n* = 15 each; 5 women per group) using a computer-generated allocation sequence with a 1:1 ratio, prepared by an independent researcher not involved in participant recruitment, data collection or analysis. Supplements and placebos were prepared in identical, opaque, coded sachets by a third party to ensure allocation concealment. Both participants and all members of the research team involved in supervision, data collection and analysis were blinded to group allocation until all data were analyzed. During the first intervention period, one group received CRE + HMB supplementation and the other received placebo. Each intervention period lasted 6 weeks and was separated by a 3-week washout phase to minimize potential carryover effects. In the second phase, participants crossed over to the alternate condition, ensuring that everyone received both treatments in a randomized sequence (Fig. 1).

The inclusion criteria were age ≥ 60 years, physical activity (engaging in at Least 150 min of moderate activity per week) and being free from severe cardiovascular, renal, hepatic or musculoskeletal disorders. For the exclusion criteria, the term ‘severe’ referred to clinically diagnosed advanced stages of these diseases (e.g. New York Heart Association Class III–IV heart failure, uncontrolled diabetes with HbA1c > 8.5%, chronic kidney disease stage ≥ 4, advanced osteoarthritis or rheumatoid arthritis with severe mobility limitation, recent osteoporotic fracture or progressive neuromuscular disease), or any other condition requiring frequent hospitalization. Individuals with mild or moderate, clinically stable forms of these conditions were eligible, provided they had medical clearance and could safely complete the exercise program [22].

All participants provided written informed consent prior to enrollment. The study was conducted in accordance with the Declaration of Helsinki, approved by the University of Burgos Ethics Committee (approval code: IR 24/2023). The trial was prospectively registered at ClinicalTrials.gov (Identifier: NCT05951439). The trial was conducted according to a pre-specified study protocol and statistical analysis plan developed before participant enrollment. The primary and secondary outcomes, analytical models and planned subgroup analyses were defined a priori, in alignment with the ClinicalTrials.gov registration (NCT05951439). During implementation, minor logistical modifications (training schedule, recruitment site and timing of assessments) were introduced to improve feasibility and adherence, without altering the primary or secondary outcomes, overall design or intervention duration. These adjustments were strictly operational and did not impact data collection or intervention fidelity. A protocol update request has been submitted to ClinicalTrials.gov to align the registry with the final procedures implemented.

Participants were recruited at a sports and fitness center in Tenerife, Spain. Prior to the start of the trial, potential candidates were approached individually by research staff during regular activities at the center and informed about the study through posters displayed in common areas. On 8 August 2024, an initial informational session was held to explain the study objectives, inclusion and exclusion criteria, intervention procedures, study timeline and assessment dates, and to address questions. Each attendee received an information sheet, a sociodemographic data form and an informed consent document.

A preliminary group of 40 individuals (15 women and 25 men) was formed. Of these, seven did not sign the informed consent because they either declined to discontinue other supplementation they were taking or did not meet other eligibility criteria. During the study, three participants withdrew before completing the second intervention period for personal reasons; no participant withdrew due to supplementation or training-related adverse effects. Mild, transient muscle soreness was experienced by some participants during the initial training sessions, which resolved without intervention.

The final sample that completed all study phases consisted of 30 participants (10 women and 20 men). The study followed a crossover design divided into three stages with four assessment points:

- Stage 1: 6 weeks of intervention, with the first control at baseline (baseline/T1, 8 November 2024) and the second at the end of the stage (T2, 20 December 2024);
- Stage 2: 3 weeks washout period (21 December 2024 to 9 January 2025);
- Stage 3: 6 weeks of intervention, with the third control at the start (T3, 10 January 2025) and the fourth at the end (T4, 21 February 2025).

With the aim of determining the effect of the supplementation, T1 and T3 values were pooled and analyzed as pre-intervention (PRE), while T2 and T4 values were pooled as post-intervention (POST). It should be noted that T1 additionally represents the pure baseline assessment prior to any intervention exposure.

### Supplementation protocol

Participants received either 3 g of CRE plus 3 g of HMB (free acid form) or 6 g of an isocaloric inulin-based placebo provided in individual sachets containing a total of 6 g of product per day. In both conditions, the supplement was mixed with yogurt or fruit juice and consumed once daily approximately 30 min before bedtime. All dietary instructions and supplementation guidelines were provided individually by a licensed dietitian-nutritionist. Supplementation packages were distributed at the T1 and T3 time point assessments, with each participant receiving a box containing 42 daily sachets (covering each 6-week intervention period). The dosage and administration protocols were based on previous studies that have demonstrated safety and efficacy in both older and athletic populations [16–18]. Compliance was monitored weekly via direct supervision, supplement counts and participant logs.

### Exercise training program

All participants followed the same individualized Integral Physical Conditioning (IPC) program during both intervention phases. The program consisted of four supervised sessions per week (60 min each), with an overall adherence rate ≥ 90%. Each session was structured into three parts: warm-up, main part and cooldown (Table 1). The primary objective was to enhance overall physical fitness and preserve functional independence in older adults, in accordance with the ACSM guidelines for exercise prescription in this population [23].

**Table 1:** Periodized Integral Physical Conditioning (IPC) program: distribution of mesocycles, microcycles, session structure and training modalities applied uniformly to all participants

Mc	mC	Type	INT (1–10)	Warm-up	Main part/session 1	Main part/session 2	Main part/session 3	Main part/session 4	Cool down
1	1	Start	5	10–12′	**MCC 1** 12 stations/3 rounds **Total time:** 40′	**STRENGTH** Legs/back/chest/core 1 EX × 2 sets × 12 reps **Total time: **30′	**MCC 2** 12 stations/3 rounds **Total time:** 30′	**STRENGTH** Arms/shoulders 1 EX × 2 sets × 12 reps Core/back care program **Total time:**30′	10′
	2	Load	6	8–10′	**B1: HIIT CORE** 10″W/20″R/4 ex/2 rounds **B2: STRENGTH** **Bodyweight pull** 3 EX × 3 sets × 10 reps **B3: MICT** 5′ run **Total time:** 45′	**MCC 3** 10 stations/3 rounds **Total time:** 35′	**STRENGTH** Legs/back/chest/shoulders/arms 2 EX × 3 sets × 10 reps **Total time:**30–40′	**MCC 4** 10 stations/3 rounds **Total time:** 35′	10′
	3	Load	7	8′	**B1: HIIT FULL BODY** 6 EX × 20″ work/30″ rest × 2 rounds **B2: STRENGTH** **Bodyweight push** 3 EX × 3 sets × 10 reps **B3: MICT** 5′ run + 5′ bike **Total time:** 45′	**STRENGTH** Legs/back/chest/shoulders/arms 1 EX × 4 sets × 8 reps **Total time:** 30–40′	**POWER** Bmed throws: 4 EX × 2 sets × 5 reps Jumps: 1EX vertical/1 EX horizontal 3 sets × 5 reps **Total time:** 40′	**STRENGTH** Arms/shoulders 2 EX × 4 sets × 8 reps Core/back care program **Total time: **45′	5′
2	4	Load	8	5–8′	**STRENGTH** Legs/back/triceps 3 EX × 4 sets × 6–8 reps **Total time**: 50′	**B1: HIIT CORE** 20´´W/20´´R/4 EX/2 rounds **B2: MCC 5** (except core) 6 stations/4 rounds **Total time:** 40´	**STRENGTH** Legs/chest/biceps 3 EX × 4 sets × 6–8 rep **Total time:** 50′	**MICT** (5′ run + 5′ rowing + 5′ elliptical trainer + 5′ bike) × 2 rounds **Total time:** 40′	5–10′
	5	Load	9	5′	**POWER** Snatch 5 sets × 5 reps/3 sets × 5 reps/2 sets × 1 rep **Total time:** 50′	**POWER** Clean & jerk 5 sets × 5 reps/3 sets × 5 reps/2sets ×1rep **Total time:** 50′	**B1: HIIT POWER** 4 EX × 10″ work/20″ rest × 2–3 rounds **B2: STRENGTH** **Leg/back** 2 EX × 3 sets × 6 reps **Total time:** 45′	**B1: HIIT GLOBAL** 4 EX × 20″ work/10″ rest × 4 rounds **B2: STRENGTH** Arms 2 EX × 3 sets × 6 reps **Total time:** 50′	5′
	6	Load	7	10′	**MCC 6** 6 stations 4–5 rounds **Total time: **45′	**MCC 7** 6 stations 4–5 rounds **Total time:** 45′	**MCC 8** 6 stations 4–5 rounds **Total time:** 45′	**MCC 9** 6 stations 4–5 rounds **Total time:** 45′	5′

*BMed* Ball medicine; *EX* Exercises; *HIIT* High-intensity interval training; *INT* Intensity; *MC* Mesocycle; *mC *Microcycle; *MICT* Moderate intensity continuous training; *MCC* Multi-component circuit

The IPC was periodized into two 6-week training blocks, each subdivided into two mesocycles of three microcycles (weeks), with a 3-week washout period without structured exercise separating the blocks. Within each block, the first microcycle served as an introductory week to facilitate familiarization and gradual load progression, while subsequent microcycles emphasized higher, individually adjusted intensities. The overall progression model was based on starting with higher volume at lower intensity to ensure safe neuromuscular adaptation and minimize injury risk, followed by a gradual reduction in volume and increase in intensity across phases to maximize gains in strength, power and functional transfer. This structured approach respects the physiology of aging, enhances safety during the early stages and prioritizes movement quality and autonomy in the advanced stages [23].

Training intensities were prescribed individually using objective and subjective measures. The training heart rate (THR) was calculated using the Karvonen formula [24]: THR = (HRmax − HRrest) × Desired intensity + HRrest. Strength loads were prescribed from the one-repetition maximum (1RM), estimated using the Brzycki formula [25]: 1RM = Weight lifted/(1.0278 − 0.0278 × Repetitions performed). To facilitate practical monitoring during training, these intensities were extrapolated to the participants’ perceived exertion (modified Borg scale). Specifically, 100% of both THR and 1RM corresponded to a rating of 10, 90% to 9, 80% to 8, 70% to 7, 60% to 6, 50% to 5 and 40% to 4, consistent with previous reports on the correlation between perceived exertion and physiological effort [26, 27]. This mapping ensured consistent regulation of exercise intensity, combining physiological calculations with real-time feedback on effort, thus achieving a common monitoring system across all protocols.

The structure of each session was as follows:

- **Warm-up (5–12 min):** Progressive mobility, balance, coordination and flexibility drills, such as joint mobilization, dynamic stretching, walking or light jogging and functional movement patterns. The primary objective was to activate the cardiometabolic system and neuromuscular pathways, preparing participants for the main training load while minimizing injury risk.
- **Main part (20–50 min):** This phase integrated different training modalities, implemented either as single-focus sessions or combined within multicomponent formats. A wide range of equipment (e.g. kettlebells, elastic bands, medicine balls, plyometric boxes, suspension systems, ropes, sleds, bikes, rowing machines and ellipticals) was used to maximize variability and adherence to functional training principles. The modalities included:
  - **Strength training:** Multi-joint and single-joint exercises targeting all major muscle groups (legs, back, chest, core, arms and shoulders) were performed using free weights, machines, resistance bands or body weight. Training loads progressed throughout the microcycles, ranging from 50 to 60% of the estimated 1RM in the introductory weeks (in accordance with ACSM guidelines [23]) to 60% and 90% in the load microcycles [28]. Participants typically performed 2 to 4 sets of 6 to 12 repetitions, with 1-to-3-min rest periods, depending on the goal. The sessions alternated between global compound exercises (e.g. squats, chest press, deadlift) and accessory or analytical movements (e.g. bicep curls, tricep extensions, lateral raises) to ensure balanced development. Strength training was carried out as a stand-alone protocol or integrated into multicomponent circuits, following a structured sequence to progressively overload different muscle groups throughout the training block.
  - **Power training:** Explosive, high-velocity movements were incorporated to enhance neuromuscular recruitment and functional performance. Exercises included medicine ball throws, plyometric jumps and ballistic lifts (e.g. snatch, clean & jerk). Intensities were typically set at 20–50% of estimated 1RM in accordance with ACSM recommendations [23], but in advanced phases, loads were progressively increased to ≥ 60–80% of 1RM, as supported by previous studies in older adults [29]. Loads were adjusted individually to maximize peak power output, identified when movement speed or jump height began to decline. Sessions generally involved 2–5 sets of 1–5 repetitions, with 30 s to 3 min rest depending on the exercise and objective.
  - **Multicomponent circuits (MCC):** Nine different MCC sessions were designed and applied uniformly to all participants throughout the intervention (Supplementary Table S1). These circuits comprised between 6 and 12 stations focusing on strength, cardiovascular endurance, speed, flexibility, mobility, balance and coordination. In this training protocol, priority was given to exercises that simultaneously developed several abilities in addition to the main one. For example, in the 12–10 station circuits, global or compound strength exercises (e.g. squats, push-ups, deadlifts) were combined with balance tasks (e.g. single-leg stance with medicine ball throw) or coordination tasks (e.g. ladder drills, zigzag runs with cones), ensuring that multiple abilities were addressed within the same station [30].
    In microcycles with two MCC sessions, both sessions complemented each other to ensure complete coverage of physical abilities. In these cases, the second session systematically increased the difficulty of balance, coordination and agility exercises (e.g. step jumps, multidirectional sprints with quick changes). In 6-station circuits, strength was prioritised, combining global exercises (e.g. bench press, leg press) with analytical work (e.g. bicep curls, leg extensions) and cardiovascular endurance exercises (e.g. bike sprints, jumping rope). Dynamic coordination was often integrated into these tasks (e.g. dribbling a ball while running).
    Strength exercises within the circuits were organised by muscle area and ordered by size, with priority given to large muscle groups (e.g. squats, chest press) over smaller ones (e.g. lateral raises, triceps extensions), so that the whole body was trained throughout the four weekly sessions of each microcycle. Cardiovascular demands were maintained between 60 and 100% of THR, with 10- to 30-s rest periods between stations and 0- to 2-min rest periods between rounds.
    Supplementary Table S1 provides a detailed description of the nine MCC sessions, including the exercise sequence, progression strategies and station design.
  - **High-intensity interval training (HIIT):** HIIT sessions alternated short bouts of high-intensity exercise with periods of rest or active recovery. Intervals typically lasted 20–60 s at 80–100% THR or ≥ 80% 1RM (for strength-power tasks), followed by 20–90 s of rest or active recovery depending on the exercise type. Sessions were structured into 4–8 work intervals per block, with 2–4 blocks per session and recovery between blocks lasting 2–3 min. Progression across microcycles was achieved by increasing the number of intervals, reducing recovery time or raising the technical/strength demand of the exercises [31].
    Four HIIT modalities were employed, designed to target complementary physical capacities and applied homogeneously to all participants:
    - **HIIT core:** Targeted trunk stabilizers (abdominals, obliques, glutes, spinal extensors) with the aim of enhancing stability, postural control and core strength through exercises such as dynamic planks, hollow rocks or Russian twists.
    - **HIIT global:** Emphasized whole-body functional movements with high metabolic demand to improve cardiovascular fitness and energy expenditure, using exercises such as burpees, thrusters, kettlebell swings or squat jumps.
    - **HIIT full body:** Integrated push, pull, lower body and core movements in balanced circuits, with the objective of simultaneously developing strength and muscular endurance while supporting hypertrophy and aerobic capacity; examples included push-ups, suspension rows (inverted rows using suspension straps), lunges and jumps.
    - **HIIT power:** Designed to enhance neuromuscular recruitment, anaerobic power and explosiveness through plyometric drills, medicine ball throws, short sprints and Olympic lifts with moderate loads.
  - **Moderate intensity continuous training (MICT):** Aerobic endurance training was implemented through continuous or interval-based activities performed at 40–60% of the training heart rate (THR). Exercises included brisk walking, stair climbing, cycling or elliptical training, adapted to individual preferences and capacities. Sessions consisted of 1–4 sets of 3–20 min, with minimal recovery between sets, depending on the prescribed duration and intensity [32]. Progression across microcycles was achieved by gradually increasing the duration of continuous bouts, introducing interval formats with higher workloads or combining modalities within the same session, while maintaining the moderate intensity range.
- **Cooldown (5–10 min):** Each session concluded with a cooldown period designed to promote recovery and restore homeostasis [33]. Activities included low-intensity movements such as light walking or mobility drills, followed by static and dynamic stretching of the major muscle groups. Additional strategies such as proprioceptive neuromuscular facilitation (PNF) stretching, myofascial release with foam rolling and guided breathing or relaxation exercises were incorporated to reduce muscle tension, facilitate flexibility and support post-exercise recovery.

### Anthropometry and body composition

Height and body mass were measured using a calibrated stadiometer and digital scale, respectively and body mass index (BMI) was calculated as weight (kg) divided by height squared (m^2^). Body composition was assessed pre- and post-intervention using a segmental multifrequency bioelectrical impedance analysis device (Tanita® MC-580; Tanita Corporation, Tokyo, Japan).

The parameters included in this study were weight, BMI, fat mass (kg), fat-free mass (kg), total muscle mass (kg), skeletal muscle mass (kg), appendicular skeletal lean mass (ALM, kg), muscle mass index (MMI), skeletal muscle index (SMI) and ALM/BMI. MMI was calculated as total muscle mass divided by height squared (kg/m^2^), and ALM/BMI as ALM (appendicular lean mass) divided by BMI (kg/m^2^). Skeletal muscle index (SMI) was calculated as appendicular skeletal muscle mass (ALM, kg) divided by height squared (m^2^). This parameter is widely used to assess sarcopenia risk and classify muscle mass adequacy in older adults, as recommended by the EWGSOP2 and other international guidelines [1].

Standard precautions were strictly followed to ensure measurement accuracy: assessments were performed in a fasting state, with bladder voiding before measurement, avoidance of alcohol and strenuous exercise in the preceding 24 h, and conducted at the same time of day under controlled hydration conditions. These procedures are consistent with recommended protocols for older adult populations and help minimize potential variability due to hydration status, recent food intake and physical activity [34].

The reliability of segmental multifrequency bioelectrical impedance analysis (BIA) devices has demonstrated test–retest coefficients of variation below 2%, consistent with previous validation studies in older adult populations [34]. This ensures high precision in tracking longitudinal changes in body composition.

### Functional strength assessment

Functional strength was evaluated at baseline and post-intervention, using a standardized battery of tests designed to assess both maximal strength and muscular endurance. All evaluations were performed after 48 h without structured exercise, and participants were instructed to avoid strenuous activities during this period to minimize fatigue effects. Assessments were conducted approximately three hours after consuming a standardized carbohydrate-rich meal (containing ~ 60% carbohydrates), ensuring a similar nutritional and energetic status among participants.

- **Handgrip strength** (kg) was measured using a digital dynamometer (Jamar® Plus +), with the participants seated and the elbow at 90° flexion. The highest value of the two maximal voluntary contractions (one per hand) was recorded [35].
- **Isometric leg and back strength** (kg) were evaluated with a hydraulic dynamometer (BASELINE®), performed in a standing position with knees slightly flexed and back straight. The participants were instructed to exert maximal force in a single pull, and the best value from two trials was used [36].
- **Isometric arm flexion strength** (kg) was assessed by using a seated resistance machine biceps curl (BASELINE®) with controlled concentric and eccentric phases. One-repetition maximum (1-RM) was estimated from submaximal attempts following standardized protocols [37].
- **30-s dumbbell arm flexion test:** Participants performed as many repetitions as possible of biceps curls using a pre-defined dumbbell weight (5 kg for women and 8 kg for men), maintaining proper form and full range of motion. The total number of complete repetitions was recorded [38].
- **The 30-s push-up tests:** were used to assess muscular endurance of the core and upper body, respectively. Participants performed as many repetitions as possible in 30 s, maintaining the correct technique without compensatory movements [39].
- **Isometric pull-up hold (seconds):** Conducted on a horizontal bar, measuring the maximum duration participants could maintain an isometric “chin-over-bar” position. Proper form was ensured by preventing excessive swinging or leg movements [38].

All tests were administered and supervised by experienced evaluators who were blinded to the supplementation conditions to ensure objectivity and consistency across the sessions.

### Nutritional evaluation

The participants were instructed to maintain their habitual dietary patterns throughout the study. They were specifically encouraged to ensure adequate intake of protein (> 1.2 g/kg body weight per day) and overall energy (~ 35–40 kcal/kg/day), as recommended for older active individuals [3].

To monitor adherence and verify that no significant dietary changes occurred between the intervention periods, two Food Frequency Questionnaires (FFQs) were administered at the end of each 6-week intervention phase. The FFQ was designed to assess the consumption frequency of 24 food items grouped into functional categories (dairy, meat, fish, cereals, fruits, vegetables, healthy fats, beverages and processed foods). This instrument was previously validated for use in physically active older adults to ensure content accuracy and relevance [40]. Analysis of the FFQ data confirmed that no significant differences in energy or macronutrient intake were present between the CRE + HMB and placebo groups.

### Statistical analysis

For the crossover analyses, T1 and T3 values were considered together as pre-intervention (PRE), and T2 and T4 values as post-intervention (POST). For baseline comparisons, only the values obtained prior to any intervention exposure (T1) were used.

All statistical analyses were performed using IBM® SPSS Statistics version 25 (IBM Corp., Armonk, NY, USA). Data are presented as means (standard deviations). Normality of the distribution was verified using the Shapiro–Wilk test.

To evaluate the overall interaction effects, a two-way repeated measures ANOVA (time × supplementation condition), with age and PRE values used as covariates to adjust for potential confounding effects, was performed for all primary outcomes. The partial eta squared (*η*^2^_p_) was used to quantify the effect size. When significant interactions or main effects were observed, Bonferroni-corrected post hoc analyses were applied to control for multiple comparisons.

Group differences at each time point (PRE and POST) were further analyzed using univariate ANOVA models, with group as a fixed factor and age as a covariate. Intra-group changes over time were assessed using one-way repeated measures ANOVA (factor: time) within each supplementation condition, again adjusting for age as a covariate to examine independent pre- and post-effects.

Linear regression analyses were used to explore the contribution of body composition changes to improvements in functional strength outcomes. For these models, delta (Δ) values were computed as relative percentage changes, calculated as [(post-intervention value – pre-intervention value)/pre-intervention value] × 100. These Δ percentages were used as predictors to account for individual pre-intervention variability.

In model 1, only supplementation condition was included as a predictor. Model 2 was additionally adjusted for age, sex and Δ skeletal muscle mass to assess independent group effects after covariate adjustment. Standardized beta coefficients (*β*), *p*-values and adjusted *R*^2^ values are reported for each model. For model 2, the presented *β* and *p*-values corresponded to the group predictor after adjustment. Collinearity diagnostics included tolerance and variance inflation factor (VIF), and the independence of residuals was checked using the Durbin–Watson statistic. Residual normality and homoscedasticity were evaluated using histogram inspection and residual plots. Statistical significance was set at *p* < 0.05.

## Results

At baseline (T1, prior to any intervention exposure), no significant differences were observed between groups in any of the studied variables (Supplementary Tables S2–S3). Similarly, at the pre-intervention (PRE) time point, there were no significant differences between the groups in any body composition parameter (all *p* > 0.05) (Table 2).

**Table 2:** Body composition parameters before and after supplementation with creatine plus HMB or placebo in older physically active adults

	Total sample (***n*** = 30)			Male (***n*** = 20)			Female (***n*** = 10)		
	CRE + HMB	PLACEBO	***p*** ***Ƞ***^2^_p_	CRE + HMB	PLACEBO	***p*** ***Ƞ***^2^_p_	CRE + HMB	PLACEBO	***p*** ***Ƞ***^2^_p_
Weight (kg)									
PRE	76.51 (13.38)	76.64 (13.89)	0.748 0.002	82.17 (11.13)	82.66 (11.66)	0.478 0.014	65.19 (10.08)	64.59 (9.63)	0.563 0.021
POST	76.75 (13.42)	76.75 (13.73)		82.48 (11.16)	82.65 (11.52)		65.31 (9.94)	64.97 (9.80)	
BMI (kg/m^2^)									
PRE	26.46 (4.19)	26.56 (4.28)	0.851 0.001	27.32 (3.70)	27.60 (3.85)	0.532 0.011	24.73 (4.77)	24.49 (4.53)	0.480 0.032
POST	26.53 (4.20)	26.61 (4.27)		27.41 (3.74)	27.59 (3.78)		24.76 (4.70)	24.66 (4.70)	
Fat mass (kg)									
PRE	17.31 (7.71)	17.03 (7.55)	**0.002** 0.158	16.64 (7.90)	16.86 (7.95)	**0.026** 0.130	18.66 (7.53)	17.39 (7.08)	**0.027** 0.130
POST	16.55 (7.89)	17.34 (7.57)		15.89 (7.97)	17.06 (7.89)		17.87 (7.97)	17.91 (7.25)	
Fat-free mass (kg)									
PRE	59.20 (10.69)	59.58 (10.66)	** < 0.001** 0.227	65.54 (6.27)	65.78 (6.52)	**0.006** 0.192	46.52 (4.20)	47.20 (4.61)	**0.007** 0.374
POST	60.22 (10.90)	59.41 (10.49)		66.59 (6.58)	65.58 (6.22)		47.48 (4.59)	47.08 (4.43)	
Total muscle mass (kg)									
PRE	56.23 (10.19)	56.61 (10.16)	** < 0.001** 0.240	62.27 (5.97)	62.51 (6.21)	**0.005** 0.203	44.15 (3.99)	44.80 (4.39)	**0.005** 0.203
POST	57.22 (10.38)	56.43 (10.01)		63.29 (6.27)	62.31 (5.94)		45.09 (4.35)	44.68 (4.22)	
Skeletal muscle mass (kg)									
PRE	33.51 (6.05)	33.74 (6.03)	** < 0.001** 0.235	37.09 (3.56)	37.25 (3.67)	**0.005** 0.198	26.34 (2.38)	26.72 (2.59)	**0.005** 0.403
POST	34.08 (6.16)	33.63 (5.94)		37.68 (3.73)	37.12 (3.51)		26.89 (2.59)	26.64 (2.51)	
Appendicular skeletal muscle mass (kg)									
PRE	24.83 (5.72)	25.07 (5.05)	**0.023** 0.090	28.20 (3.59)	28.44 (3.83)	**0.008** 0.177	18.10 (1.70)	18.42 (1.80)	**0.033** 0.217
POST	25.28 (5.84)	24.99 (5.71)		28.71 (3.65)	28.31 (3.66)		18.32 (1.83)	18.35 (1.88)	
Muscle mass index (kg/m^2^)									
PRE	14.60 (3.52)	15.27 (3.75)	**0.012** 0.107	14.70 (3.06)	15.30 (3.49)	**0.033** 0.120	14.40 (4.50)	15.20 (4.41)	0.141 0.130
POST	15.27 (3.63)	15.07 (3.59)		15.45 (3.26)	15 (3.22)		14.90 (4.45)	15.20 (4.41)	
Skeletal muscle index (kg/m^2^)									
PRE	8.51 (1.52)	8.64 (1.59)	**0.008** 0.120	9.33 (0.97)	9.48 (1.07)	**0.002** 0.237	6.86 (0.96)	6.96 (1.02)	**0.022** 0.285
POST	8.67 (1.58)	8.60 (1.55)		9.52 (1.07)	9.42 (1.01)		6.99 (0.97)	6.96 (1.03)	
ALM/BMI									
PRE	0.94 (0.19)	0.98 (0.18)	**0.002** 0.154	1.04 (0.14)	1.04 (0.14)	**0.023** 0.136	0.74 (0.09)	0.76 (0.09)	**0.018** 0.303
POST	0.95 (0.19)	0.94 (0.18)		1.05 (0.14)	1.03 (0.14)		0.75 (0.10)	0.75 (0.10)	

Data are presented as mean (standard deviation)

*p*: Two-way repeated measures ANOVA (time × supplementation condition), adjusted for age and pre-intervention (PRE) value were used to assess interaction effects. Post hoc comparisons were Bonferroni-corrected

*η*^2^_p_: partial eta squared effect size

*Significant difference between groups at the same time point (*p* < 0.05) by univariate ANOVA with group as a fixed factor, adjusted for age and PRE value

$Significant within-group difference from pre-intervention (*p* < 0.05) by one-way repeated measures ANOVA (factor: time), adjusted for age and PRE value

PRE corresponds to the data of T1 and T3 (pre-intervention) and POST to the data of T2 and T4 (post-intervention)

After the intervention, significant time × group interaction effects were found for fat mass, fat-free mass, total muscle mass, skeletal muscle mass, appendicular skeletal muscle mass, muscle mass index, skeletal muscle index and ALM/BMI (all *p* < 0.05), indicating differential trends over time between the groups.

In the total sample, the CRE + HMB group showed reductions in fat mass (from 17.31 ± 7.71 to 16.55 ± 7.89 kg) and body fat percentage (from 22.34 ± 8.31% to 21.26 ± 8.73%), together with slight numerical increases in fat-free mass (from 59.20 ± 10.69 to 60.22 ± 10.90 kg), total muscle mass (from 56.23 ± 10.19 to 57.22 ± 10.38 kg), skeletal muscle mass (from 33.51 ± 6.05 to 34.08 ± 6.16 kg) and appendicular skeletal muscle mass (from 24.83 ± 5.72 to 25.28 ± 5.84 kg). In contrast, the placebo group exhibited slight increases in fat mass and body fat percentage, while muscle-related parameters remained stable or decreased. However, these changes did not reach statistical significance in within-group comparisons (*p* > 0.05).

When stratified by sex, men in the CRE + HMB group showed numerical increases in fat-free mass (from 65.54 ± 6.27 to 66.59 ± 6.58 kg), total muscle mass (from 62.27 ± 5.97 to 63.29 ± 6.27 kg), skeletal muscle mass (from 37.09 ± 3.56 to 37.68 ± 3.73 kg) and appendicular skeletal muscle mass (from 28.20 ± 3.59 to 28.71 ± 3.65 kg), along with numerical reductions in fat mass (from 16.64 ± 7.90 to 15.89 ± 7.97 kg) and body fat percentage (from 19.62 ± 7.49 to 18.64 ± 7.69%). However, no statistically significant within-group or between-group differences were observed. Conversely, the placebo group exhibited a slight increase in fat mass and minimal changes or reductions in muscle-related parameters. Among women, similar improvements in lean mass and reductions in fat mass were observed, but no significant differences were detected within or between the groups.

The partial eta-squared effect sizes (*η*^2^_p_) for these parameters ranged from small to moderate, suggesting a potential intervention effect despite the lack of statistically significant post hoc differences.

At pre-intervention (PRE) time point, no significant differences were observed between the groups in most strength-related variables, except for crunches, where the placebo group showed significantly higher values in the total sample and in women (*p* < 0.05) (Table 3).

**Table 3:** Functional strength parameters before and after supplementation with creatine plus HMB or placebo in older physically active adults

	Total sample (*N* = 30)			Male (*N* = 20)			Female (*N* = 10)		
Group/period	CRE + HMB	PLACEBO	*p* ***Ƞ***^2^_p_	CRE + HMB	PLACEBO	***p*** ***Ƞ***^2^_p_	CRE + HMB	PLACEBO	*p* ***Ƞ***^2^_p_
Grip strength (kg)									
PRE	33.90 (9.73)	33.59 (8.49)	**0.010** 0.112	39.03 (7.41)	38.17 (6.16)	**0.022** 0.138	23.65 (3.80)	24.43 (3.53)	0.281 0.0.72
POST	36.96 (10.38)	34.52 (8.37)		42.06 (8.38)	38.73 (6.79)		26.76 (5.02)	26.10 (3.18)	
Leg and back dyna strength (kg)									
PRE	85.62 (26.00)	89.6 (28.57)	**0.004** 0.140	96.81 (24.01)	101.35 (26.71)	**0.024** 0.134	63.24 (11.29)	66.09 (14.23)	**0.049** 0.208
POST	106.81 (34.12)	91.70 (24.56)*		121.70 (31.69)$	103.30 (19.69)*		77.03 (12.34)$	68.5 (15.09)*	
Arms flexion dyna strength (kg)									
PRE	39.26 (15.13)	40.74 (14.39)	** < 0.001** 0.269	45.52 (14.37)	46.87 (13.11)	** < 0.001** 0.317	26.75 (6.41)	28.49 (7.34)	** < 0.050** 0.219
POST	50.97 (20.44)$	41.34 (16.50)		59.20 (19.11)$	47.14 (15.00)*		34.51 (11.10)$	29.75 (13.37)	
Dumbbell arm flexion (30 s)									
PRE	28.2 (5.55)	31.23 (9.00)	** < 0.001** 0.289	28.60 (6.03)	31.70 (8.74)	** < 0.001** 0.316	27.40 (4.64)	30.30 (9.91)	**0.045** 0.229
POST	39.83 (10.65)	32.07 (7.56)*		40.40 (10.03)	33.05 (7.84)*		38.70 (12.29)	30.10 (6.93)*	
Crunches (30 se)									
PRE	26.33 (5.72)	28.8 (8.87)*	**0.001** 0.404	26.45 (6.11)	27.95 (8.80)	**0.002** 0.229	26.1 (5.15)	30.5 (9.24)	**0.004** 0.276
POST	34.93 (7.43)$	29.83 (7.41)		34.80 (7.87)$	35 (7.42)		34.8 (7.87)$	30.6 (6.60)	
Push-up (30 s)									
PRE	14.93 (6.77)	17.5 (7.93)	** < 0.001** 0.404	16.55 (6.89)	19.5 (8.19)	** < 0.001** 0.440	11.70 (5.49)	13.50 (5.83)	**0.004** 0.407
POST	21.83 (8.38)$	17.13(8.71)		25.25 (7.16)	19.7 (9.20)		15.00 (6.36)	12.00 (4.71)	
Isometric pull-ups (s)									
PRE	15.01 (13.46)	14.99 (11.52)	**0.001** 0.190	18.53 (14.38)	16.72 (11.34)	**0.003** 0.226	7.97 (8.03)	11.54 (11.69)	0.154 0.150
POST	21.09 (15.08)$	15.22 (11.73)		25.34 (15.42)	17.03 (12.23)*		12.58 (10.51)	11.61 (10.28)	

Data are presented as mean (standard deviation)

*p*: Two-way repeated measures ANOVA (time × supplementation condition) adjusted for age and pre-intervention (PRE) value were used to assess interaction effects. Post hoc comparisons were Bonferroni-corrected

*η*^2^_p_: partial eta squared effect size

*Significant difference between groups at the same time point (*p* < 0.05) by univariate ANOVA with group as a fixed factor, adjusted for age and PRE value

$Significant within-group difference from pre-intervention (*p* < 0.05) by one-way repeated measures ANOVA (factor: time), adjusted for age and PRE value

PRE corresponds to the data of T1 and T3 (pre-intervention) and POST to the data of T2 and T4 (post-intervention)

After the intervention, significant time × group interaction effects were observed for grip strength, leg and back strength, arm flexion strength, dumbbell arm flexion (30 s), push-ups, crunches and isometric pull-ups (all *p* < 0.05), indicating a differential response between the groups.

In the total sample, participants in the CRE + HMB group showed substantial improvements in all strength and endurance tests, with increases in grip strength (from 33.90 ± 9.73 to 36.96 ± 10.38 kg), leg and back strength (from 85.62 ± 26.00 to 106.81 ± 34.12 kg and arms flexion strength (from 39.26 ± 15.13 to 50.97 ± 20.44 kg). Additionally, marked improvements were seen in dynamic endurance tests: dumbbell arm flexion (from 28.2 ± 5.55 to 39.83 ± 10.65 reps), crunches (from 26.33 ± 5.72 to 34.93 ± 7.43 reps), push-ups (from 14.93 ± 6.77 to 21.83 ± 8.38 reps) and isometric pull-ups (from 15.01 ± 13.46 to 21.09 ± 15.08 s).

Conversely, the placebo group showed minimal or no improvement; in some cases, performance remained stable or tended to decline slightly post-intervention. For example, leg and back strength changed only marginally (from 89.6 ± 28.57 to 91.70 ± 24.56 kg), and similar trends were seen across other parameters.

In men, the CRE + HMB group exhibited significant improvements in leg and back strength (from 96.81 ± 24.01 to 121.70 ± 31.69 kg), arms flexion strength (from 45.52 ± 14.37 to 59.20 ± 19.11 kg) and crunches (from 26.45 ± 6.11 to 34.80 ± 7.87 reps), while the placebo group remained largely unchanged or showed minimal gains.

In women, although numerical improvements were also evident in the CRE + HMB group (e.g. leg and back strength from 63.24 ± 11.29 to 77.03 ± 12.34 kg), the placebo group generally maintained or slightly worsened their pre-intervention values, with no significant post-intervention differences observed between groups.

The partial eta-squared values (*η*^2^_p_) ranged from small to large, supporting a relevant effect of the intervention on functional strength outcomes.

Regression analyses showed that in model 1, group allocation (CRE + HMB) significantly predicted changes in most functional strength outcomes, including grip strength (*β* = 0.307, *p* = 0.017), leg/back strength (*β* = 0.373, *p* = 0.003), arm flexion strength (*β* = 0.520, *p* < 0.001), 30-s dumbbell flexion (*β* = 0.563, *p* < 0.001), 30-s crunches (*β* = 0.450, *p* < 0.001), 30-s push-ups (*β* = 0.646, *p* < 0.001) and isometric pull-up hold time (*β* = 0.411, *p* = 0.001). The explained variance (adjusted *R*^2^) ranged from 7.9 for grip strength to 40.8% for push-ups, indicating the substantial contribution of supplementation to these improvements (Table 4).

**Table 4:** Summary of regression models evaluating the effect of supplementation and body composition on functional strength outcomes

	**Model 1**			**Model 2**			
**Outcome**	***β***	***p***	**Adj. *R***^**2**^	***β***	***p***	**Adj. *R***^**2**^	**Effect interpretation**
Δ Grip strength	0.307	0.017	0.079	0.223	0.111	0.146	Possible mediation
Δ Leg/back strength	0.373	0.003	0.124	0.344	0.018	0.099	Direct effect
Δ Arm flexion strength	0.52	< 0.001	0.257	0.472	0.001	0.231	Direct robust effect
Δ Dumbbell flexion (30 s)	0.563	< 0.001	0.305	0.557	< 0.001	0.278	Direct robust effect
Δ Crunches (30 s)	0.45	< 0.001	0.189	0.441	0.002	0.145	Direct robust effect
Δ Push-ups (30 s)	0.646	< 0.001	0.408	0.631	< 0.001	0.417	Strong direct effect
ΔIsometric pull-up hold	0.411	0.001	0.154	0.35	0.014	0.197	Direct effect, age relevant

Δ values for all variables were calculated as [(POST – PRE)/PRE] × 100

Model 1: supplementation group as the sole predictor

Model 2: supplementation group adjusted for age, sex and Δ skeletal muscle mass

The reported *β*, *p* and Adj. *R*^2^ values in model 2 refer specifically to the group predictor after adjustment. *β*: standardized regression coefficient; Adj. *R*^2^: adjusted coefficient of determination. The “effect interpretation” column indicates whether the supplementation effect is direct or potentially mediated

In model 2, after adjusting for age, sex and Δ skeletal muscle mass, the effect of group allocation remained significant for leg/back strength (*β* = 0.344, *p* = 0.018), arm flexion strength (*β* = 0.472, *p* = 0.001), 30-s dumbbell flexion (*β* = 0.557, *p* < 0.001), 30-s crunches (*β* = 0.441, *p* = 0.002), 30-s push-ups (*β* = 0.631, *p* < 0.001) and isometric pull-up hold time (*β* = 0.350, *p* = 0.014), but not for grip strength (*β* = 0.223, *p* = 0.111).

Notably, for isometric pull-up hold time, age was also a significant predictor (*β* = 0.262, *p* = 0.040), indicating that younger participants tended to show greater improvement. Overall, these findings support a robust and predominantly direct effect of CRE + HMB supplementation on functional strength and endurance outcomes, with grip strength potentially being influenced by additional mediating factors.

## Discussion

This study aimed to demonstrate that joint supplementation with creatine monohydrate plus β-hydroxy-β-methylbutyrate (CRE + HMB), combined with an Integral Physical Conditioning (IPC) program, enhances physical capacity in older adults, particularly through mechanisms not solely dependent on muscle hypertrophy. This integrated strategy may support independence and mitigate loss of functional capacity, which is commonly associated with aging.

The primary finding was that CRE + HMB supplementation significantly improved multiple functional strength outcomes in physically active older adults, including leg strength, arm flexion strength, upper-body endurance (dumbbell flexion, push-ups, isometric hold) and core endurance (crunches). Regression analyses revealed that these improvements were largely independent of changes in skeletal muscle mass or sarcopenia-related indices (e.g. SMI), apart from grip strength. These findings suggest that functional enhancements are driven not only by muscle hypertrophy but also by neuromuscular adaptations.

Possible neuromuscular mechanisms include improved motor unit recruitment efficiency, enhanced neural drive and improved coordination and synchronization of muscle contractions. Such adaptations could translate into superior performance in daily activities, emphasizing the importance of addressing both muscular and neural components when designing interventions for older adults. These results underscore the multifactorial nature of strength development and highlight the potential of CRE + HMB supplementation combined with IPC to support a more comprehensive strategy for healthy aging.

These findings align with previous evidence supporting the efficacy of creatine supplementation in older adults, where it has been shown to enhance muscle strength and lean mass when combined with resistance training [8, 41]. Mechanistically, creatine increased intramuscular phosphocreatine content, facilitated rapid ATP synthesis, activated anabolic pathways (e.g. mTOR), reduced protein breakdown, induced cell volumization and attenuated oxidative stress and inflammation [11, 12]. Collectively, these multifactorial mechanisms may explain the greater improvements observed in both muscle quality and functional capacity in the CRE + HMB group.

In contrast, HMB attenuates muscle protein breakdown primarily by downregulating the ubiquitin–proteasome pathway and promoting anabolic signaling via mTOR [13, 14]. HMB may also improve membrane integrity and reduce muscle damage, particularly during periods of high catabolic stress or disuse [14, 15]. HMB may also improve membrane integrity and reduce muscle damage, particularly during periods of high catabolic stress or muscle disuse.

This study extends previous athlete-based research on CRE + HMB co-supplementation [16–18], which showed additive benefits in strength, body composition and recovery. Fernández-Landa et al. (2020) demonstrated that this combination improved performance and modulated anabolic–catabolic hormone profiles in elite endurance athletes [16]. Our findings suggest that similar synergistic benefits may translate into older adults, supporting its potential as a targeted intervention to enhance function and independence.

Notably, the strength gains in this study were not fully explained by an increase in muscle mass. Although hypertrophy is traditionally viewed as the primary mechanism for strength improvement [19], emerging evidence highlights the critical role of neural adaptations, including increased motor unit recruitment, firing rates, improved intermuscular coordination and reduced antagonist coactivation [20, 23]. These neural mechanisms, previously documented in athletes and younger adults [42, 43], have also been described in older adults undergoing resistance training [44]. Our results suggest that CRE + HMB supplementation may potentiate such adaptations, leading to enhanced muscle quality and contractile efficiency, consistent with previous observations in athletes [16–18].

Improvements in upper-body endurance (e.g. push-ups, dumbbell flexion and isometric hold) also indicate metabolic adaptations, including increased phosphocreatine stores, improved ATP resynthesis and better energy-buffering capacity [9, 10]. These changes may help delay fatigue, reduce metabolite accumulation and improve contractile efficiency during repeated effort [45, 46]. Such adaptations are particularly relevant for daily tasks in older adults, such as carrying groceries or rising from a chair, which require repeated upper-body exertion [47].

Additionally, the regression models indicated that improvements in isometric hold time were partially influenced by age, with younger participants experiencing greater gains. This is consistent with evidence showing age-related declines in neuromuscular plasticity due to reduced motor unit remodeling, lower motor neuron excitability and compromised neuromuscular junction integrity [48, 49]. These findings reinforce the value of early interventions to maximize functional reserve and delay age-related decline in muscle performance.

From a clinical perspective, CRE + HMB supplementation combined with IPC represents a promising strategy to improve muscle strength and functional capacity in older adults, potentially enhancing independence, reducing fall risk and preserving the quality of life. As sarcopenia is now recognized as a multifactorial geriatric syndrome involving both quantitative (mass) and qualitative (function) deficits [1], interventions addressing both dimensions are particularly valuable. Dual-action approaches may also reduce healthcare costs by lowering the incidence of disability, institutionalization and fall-related injuries [2, 50].

While this study benefits from a rigorous randomized, double-blind crossover design that minimizes inter-individual variability through comprehensive functional assessments, several limitations merit consideration. Although the total sample (*n* = 30) achieved adequate power (~ 78.4%) for the main analyses, with effect sizes (*η*^2^*p* = 0.17–0.42) exceeding the a priori assumption (*f* = 0.25) and excellent retention (10% attrition), the sex-stratified subgroups (20 men and 10 women) were underpowered for detecting sex-specific effects, and these analyses should therefore be interpreted with caution. In addition, the a priori sample size calculation was based on grip strength as a clinically representative endpoint and did not formally adjust for the presence of multiple correlated co-primary endpoints. Although endpoint correlations and the efficiency of the crossover design mitigate this concern, the absence of multiplicity correction may have reduced the effective statistical power. Future trials with larger and more diverse samples should incorporate conservative approaches, such as multivariate power calculations or family-wise error rate adjustments, to further strengthen methodological rigor.

Additionally, while bioelectrical impedance analysis (BIA) offers a practical and non-invasive method for estimating body composition, it has inherent limitations in capturing subtle changes in muscle mass compared to gold-standard techniques, such as dual-energy X-ray absorptiometry (DXA). Furthermore, the relatively short duration of the intervention and absence of long-term follow-up limits the conclusions regarding the sustainability of the observed effects. Another limitation is the absence of separate supplementation arms (creatine or HMB alone), which precludes determination of the individual contributions of each supplement to the observed outcomes.

As noted in methods, minor logistical adjustments (training schedules, recruitment procedures) were implemented during the trial to optimize feasibility. While these were formally documented through a ClinicalTrials.gov protocol update (NCT05951439) and did not affect primary outcomes, they underscore the balance between methodological rigor and practical implementation in exercise–nutrition interventions.

It is important to note as a limitation of the study that the absence of direct neuromuscular measurements (e.g. electromyography and muscle biopsies) prevented confirmation of the neural adaptations proposed to underlie the functional improvements. Future studies should incorporate these metrics, as well as molecular biomarkers such as microRNAs related to muscle plasticity, to better elucidate the pathways mediating these effects. Nevertheless, our decision to evaluate this combination was supported by previous evidence suggesting potential synergistic effects when CRE and HMB were co-administered [16–18], reinforcing the practical relevance of assessing this combined strategy as a single intervention in real-world settings.

From a practical perspective, combining CRE + HMB supplementation with exercise is a promising strategy for counteracting age-related declines in functional capacity. This approach could potentially reduce the risk of falls, enhance independence and improve quality of life in older adults. This approach addresses the quantitative (muscle mass) and qualitative (functional performance) aspects of sarcopenia, in line with current clinical guidelines and public health priorities for promoting healthy ageing.

While our findings provide novel insights, future studies should aim to confirm these results in larger, more diverse populations of older people. They should also explore the underlying neuromuscular mechanisms in greater depth, for example motor unit behaviour, neural drive and neuromuscular junction integrity. Furthermore, they should investigate long-term sustainability and safety. Evaluating outcomes such as mobility, balance and inflammatory or molecular biomarkers (e.g. microRNAs) would also support the practical implementation and clinical translation of these findings.

## Conclusion

The present study demonstrated that 6 weeks of supplementation with 3 g per day of creatine monohydrate (CRE) and 3 g per day of β-hydroxy-β-methylbutyrate (HMB), alongside an integral physical conditioning (IPC) program significantly improved functional strength and muscular endurance in physically active older adults. Notably, these improvements were largely independent of changes in skeletal muscle mass, indicating that neuromuscular and functional adaptations were the predominant contributors.

These findings suggest that combined nutritional and exercise interventions are an effective strategy for enhancing functional capacity, promoting autonomy and fostering healthy ageing. However, future studies are needed to confirm these results in larger and more diverse populations, explore the underlying mechanisms in greater detail and evaluate long-term outcomes in order to establish the clinical applicability of this approach.

## Supplementary Information

Below is the link to the electronic supplementary material.

ESM 1 (DOCX 47.6 KB)

## References

[1] Cruz-Jentoft AJ, Bahat G, Bauer J, Boirie Y, Bruyère O, Cederholm T, et al. Sarcopenia: revised European consensus on definition and diagnosis. Age Ageing. 2019;48(1):16–31. 10.1093/ageing/afy169. 30312372 10.1093/ageing/afy169 PMC6322506

[2] Beaudart C, Zaaria M, Pasleau F, Reginster JY, Bruyère O. Health outcomes of sarcopenia: a systematic review and meta-analysis. PLoS ONE. 2017;12(1):e0169548. 10.1371/journal.pone.0169548. 28095426 10.1371/journal.pone.0169548 PMC5240970

[3] Bauer J, Biolo G, Cederholm T, Cesari M, Cruz-Jentoft AJ, Morley JE, et al. Evidence-based recommendations for optimal dietary protein intake in older people: a position paper from the PROT-AGE study group. J Am Med Dir Assoc. 2013;14(8):542–59. 10.1016/j.jamda.2013.05.021. 23867520 10.1016/j.jamda.2013.05.021

[4] Fragala MS, Cadore EL, Dorgo S, Izquierdo M, Kraemer WJ, Peterson MD, et al. Resistance training for older adults: position statement from the National Strength and Conditioning Association. J Strength Cond Res. 2019;33(8):2019–52. 10.1519/JSC.0000000000003230. 31343601 10.1519/JSC.0000000000003230

[5] Peterson MD, Rhea MR, Sen A, Gordon PM. Resistance exercise for muscular strength in older adults: a meta-analysis. Ageing Res Rev. 2010;9(3):226–37. 10.1016/j.arr.2010.03.004. 20385254 10.1016/j.arr.2010.03.004 PMC2892859

[6] Breen L, Phillips SM. Skeletal muscle protein metabolism in the elderly: interventions to counteract the “anabolic resistance” of ageing. Nutr Metab (Lond). 2011;8:68. 10.1186/1743-7075-8-68. 21975196 10.1186/1743-7075-8-68 PMC3201893

[7] Cadore EL, Rodríguez-Mañas L, Sinclair A, Izquierdo M. Effects of different exercise interventions on risk of falls, gait ability, and balance in physically frail older adults: a systematic review. Rejuvenation Res. 2013;16(2):105–14. 10.1089/rej.2012.1397. 23327448 10.1089/rej.2012.1397 PMC3634155

[8] Dos Santos EEP, de Araújo RC, Candow DG, Forbes SC, Guijo JA, de Almeida Santana CC, et al. Efficacy of creatine supplementation combined with resistance training on muscle strength and muscle mass in older females: a systematic review and meta-analysis. Nutrients. 2021;13(11):3757. 10.3390/nu13113757. 34836013 10.3390/nu13113757 PMC8619193

[9] Kreider RB, Kalman DS, Antonio J, Ziegenfuss TN, Wildman R, Collins R, et al. International society of sports nutrition position stand: safety and efficacy of creatine supplementation in exercise, sport, and medicine. J Int Soc Sports Nutr. 2017;14:18. 10.1186/s12970-017-0173-z. 28615996 10.1186/s12970-017-0173-z PMC5469049

[10] Rawson ES, Volek JS. Effects of creatine supplementation and resistance training on muscle strength and weightlifting performance. J Strength Cond Res. 2003;17(4):822–31. 10.1519/1533-4287(2003)017<0822:eocsar>2.0.co;2. 14636102 10.1519/1533-4287(2003)017<0822:eocsar>2.0.co;2

[11] Safdar A, Yardley NJ, Snow R, Melov S, Tarnopolsky MA. Global and targeted gene expression and protein content in skeletal muscle of young men following short-term creatine monohydrate supplementation. Physiol Genomics. 2008;32(2):219–28. 10.1152/physiolgenomics.00157.2007. 17957000 10.1152/physiolgenomics.00157.2007

[12] Santos RVT, Bassit RA, Caperuto EC, Costa Rosa LFBP. The effect of creatine supplementation upon inflammatory and muscle soreness markers after a 30 km race. Life Sci. 2004;75(16):1917–24. 10.1016/j.lfs.2003.11.036. 15306159 10.1016/j.lfs.2003.11.036

[13] Holeček M. Beta-hydroxy-beta-methylbutyrate supplementation and skeletal muscle in healthy and muscle-wasting conditions. J Cachexia Sarcopenia Muscle. 2017;8(4):529–41. 10.1002/jcsm.12208. 28493406 10.1002/jcsm.12208 PMC5566641

[14] Wilkinson DJ, Hossain T, Hill DS, Phillips BE, Crossland H, Williams J, et al. Effects of leucine and its metabolite β-hydroxy-β-methylbutyrate on human skeletal muscle protein metabolism. J Physiol. 2013;591(11):2911–23. 10.1113/jphysiol.2013.253203. 23551944 10.1113/jphysiol.2013.253203 PMC3690694

[15] Berton L, Bano G, Carraro S, Veronese N, Pizzato S, Bolzetta F, et al. Effect of oral beta-hydroxy-beta-methylbutyrate (HMB) supplementation on physical performance in healthy old women over 65 years: an open label randomized controlled trial. PLoS ONE. 2015;10(11):e0141757. 10.1371/journal.pone.0141757. 26529601 10.1371/journal.pone.0141757 PMC4631374

[16] Fernández-Landa J, Calleja-González J, León-Guereño P, Caballero-García A, Córdova A, Mielgo-Ayuso J. Effect of the combination of creatine monohydrate plus HMB supplementation on sports performance, body composition, markers of muscle damage and hormone status: a systematic review. Nutrients. 2019;11(10):2528. 10.3390/nu11102528. 31635165 10.3390/nu11102528 PMC6835217

[17] Fernández-Landa J, Fernández-Lázaro D, Calleja-González J, Caballero-García A, Martínez AC, León-Guereño P, et al. Effect of ten weeks of creatine monohydrate plus HMB supplementation on athletic performance tests in elite male endurance athletes. Nutrients. 2020;12(1):193. 10.3390/nu12010193. 31936727 10.3390/nu12010193 PMC7019716

[18] Fernández-Landa J, Fernández-Lázaro D, Calleja-González J, Caballero-García A, Córdova A, León-Guereño P, et al. Long-term effect of combination of creatine monohydrate plus β-hydroxy β-methylbutyrate (HMB) on exercise-induced muscle damage and anabolic/catabolic hormones in elite male endurance athletes. Biomolecules. 2020;10(1):140. 10.3390/biom10010140. 31952174 10.3390/biom10010140 PMC7022312

[19] Schoenfeld BJ. The mechanisms of muscle hypertrophy and their application to resistance training. J Strength Cond Res. 2010;24(10):2857–72. 10.1519/jsc.0b013e3181e840f3. 20847704 10.1519/JSC.0b013e3181e840f3

[20] Del Vecchio A, Casolo A, Negro F, Scorcelletti M, Bazzucchi I, Enoka R, et al. The increase in muscle force after 4 weeks of strength training is mediated by adaptations in motor unit recruitment and rate coding. J Physiol. 2019;597(7):1873–87. 10.1113/jp277250. 30727028 10.1113/JP277250 PMC6441907

[21] Jones B, Kenward MG. Design and analysis of cross-over trials. 3rd ed. Boca Raton: CRC Press; 2014. 10.1201/B17537.

[22] Garber CE, Blissmer B, Deschenes MR, Franklin BA, Lamonte MJ, Lee IM, et al. Quantity and quality of exercise for developing and maintaining cardiorespiratory, musculoskeletal, and neuromotor fitness in apparently healthy adults: guidance for prescribing exercise. Med Sci Sports Exerc. 2011;43(7):1334–59. 10.1249/mss.0b013e318213fefb. 21694556 10.1249/MSS.0b013e318213fefb

[23] Aagaard P, Simonsen EB, Andersen JL, Magnusson P, Dyhre-Poulsen P. Increased rate of force development and neural drive of human skeletal muscle following resistance training. J Appl Physiol. 2002;93:1318–26. 10.1152/japplphysiol.00283.2002 10.1152/japplphysiol.00283.2002 12235031

[24] Karvonen J, Vuorimaa T. Heart rate and exercise intensity during sports activities: practical application. Sports Med. 1988;5(5):303–11. 10.2165/00007256-198805050-00002. 3387734 10.2165/00007256-198805050-00002

[25] Brzycki M. Strength testing—predicting a one-rep max from reps-to-fatigue. J Phys Educ Recreat Dance. 1993;64(1):88–90. 10.1080/07303084.1993.10606684.

[26] Lea JWD, O’Driscoll JM, Hulbert S, et al. Convergent validity of ratings of perceived exertion during resistance exercise in healthy participants: a systematic review and meta-analysis. Sports Med. 2022;8(1):2. 10.1186/s40798-021-00386-8. 10.1186/s40798-021-00386-8 PMC8742800 35000021

[27] Helms ER, Kwan K, Sousa CA, Cronin JB, Storey AG, Zourdos MC. Methods for regulating and monitoring resistance training. J Hum Kinet. 2020;74:23–42. 10.2478/hukin-2020-0011. 33312273 10.2478/hukin-2020-0011 PMC7706636

[28] Tøien T, Berg OK, Modena R, Brobakken MF, Wang E. Heavy strength training in older adults: implications for health, disease and physical performance. J Cachexia Sarcopenia Muscle. 2025;16(2):e130804. 10.1002/jcsm.13804. 10.1002/jcsm.13804 PMC12003923 40241440

[29] De Vos NJ, Singh NA, Ross DA, Stavrinos TM, Orr R, Singh MAF. Optimal load for increasing muscle power during explosive resistance training in older adults. J Gerontol A Biol Sci Med Sci. 2005;60(5):638–47. 10.1093/gerona/60.5.638. 15972618 10.1093/gerona/60.5.638

[30] Labata-Lezaun N, González-Rueda V, Llurda-Almuzara L, López-de-Celis C, Rodríguez-Sanz J, Bosch J, et al. Effectiveness of multicomponent training on physical performance in older adults: a systematic review and meta-analysis. Arch Gerontol Geriatr. 2023;104:104838. 10.1016/j.archger.2022.104838. 36272227 10.1016/j.archger.2022.104838

[31] Sert H, Gulbahar Eren M, Gurcay B, et al. The effectiveness of a high-intensity interval exercise on cardiometabolic health and quality of life in older adults: a systematic review and meta-analysis. BMC Sports Sci Med Rehabil. 2025;17:128. 10.1186/s13102-025-01176-5. 40413509 10.1186/s13102-025-01176-5 PMC12102952

[32] Netz Y, Wu MJ, Becker BJ, Tenenbaum G. Physical activity and psychological well-being in advanced age: a meta-analysis of intervention studies. Psychol Aging. 2005;20(2):272–84. 10.1037/0882-7974.20.2.272. 16029091 10.1037/0882-7974.20.2.272

[33] Van Hooren B, Peake JM. Do we need a cool-down after exercise? A narrative review of the psychophysiological effects and the effects on performance, injuries and the long-term adaptive response. Sports Med. 2018;48(7):1575–95. 10.1007/s40279-018-0916-2. 29663142 10.1007/s40279-018-0916-2 PMC5999142

[34] Kim M, Shinkai S, Murayama H, Mori S. Comparison of segmental multifrequency bioelectrical impedance analysis with dual-energy X-ray absorptiometry for the assessment of body composition in a community-dwelling older population. Geriatr Gerontol Int. 2015;15:1013–22. 10.1111/ggi.12384 10.1111/ggi.12384 25345548

[35] Roberts HC, Denison HJ, Martin HJ, Patel HP, Syddall H, Cooper C, Sayer AA. A review of the measurement of grip strength in clinical and epidemiological studies: towards a standardised approach. Age Ageing. 2011;40:423–9. 10.1093/ageing/afr051 10.1093/ageing/afr051 21624928

[36] Yang S, Wu W, Zhang C, Wang D, Chen C, Tang Y, Li K, Xu J, Luo F. Reliability and validity of three isometric back extensor strength assessments with different test postures. J Int Med Res.2019;48:300060519885268. 10.1177/0300060519885268 10.1177/0300060519885268 PMC7607202 31698974

[37] Romero-Franco N, Fernández-Domínguez JC, Montaño-Munuera JA, Romero-Franco J, Jiménez-Reyes P. Validity and reliability of a low-cost dynamometer to assess maximal isometric strength of upper limb. J Sports Sci. 2019;37:1787–93. 10.1080/02640414.2019.1594570 10.1080/02640414.2019.1594570 30897030

[38] Clemons JM. Construct validity of a modification of the flexed arm hang test. J Strength Cond Res. 2014;28:3523–30. 10.1519/jsc.0000000000000601 10.1519/JSC.0000000000000601 24983850

[39] Kellner P, Neubauer J, Polách M. Objectivity of push-up tests and technique assessment. J Phys Educ Sport. 2021;21:1629–34.

[40] Martin-Moreno JM, Boyle P, Gorgojo L, Maisonneuve P, Fernandez-Rodriguez JC, Salvini S, Willett WC. Development and validation of a food frequency questionnaire in Spain. Int J Epidemiol. 1993;22:512–9. 8359969 10.1093/ije/22.3.512

[41] Devries MC, Phillips SM. Creatine supplementation during resistance training in older adults: a meta-analysis. Med Sci Sports Exerc. 2014;46:1194–203. 10.1249/mss.0000000000000220 10.1249/MSS.0000000000000220 24576864

[42] Brownstein CG, Casolo A, Del Vecchio A, Ansdell P. The knowns and unknowns of neural adaptations to resistance training. Eur J Appl Physiol. 2021;121:675–85. 10.1007/s00421-020-04567-3 10.1007/s00421-020-04567-3 PMC7892509 33355714

[43] Unhjem R, Lundestad R, Fimland MS, Mosti MP, Wang E. Strength training-induced responses in older adults: attenuation of descending neural drive with age. Age (Dordr). 2015;37:47. 10.1007/s11357-015-9784-y 10.1007/s11357-015-9784-y PMC4418975 25940749

[44] Suetta C, Aagaard P, Rosted A, Jakobsen AK, Duus B, Kjaer M, Magnusson SP. Training-induced changes in muscle CSA, muscle strength, EMG, and rate of force development in elderly subjects after long-term unilateral disuse. J Appl Physiol. 2004;97:1954–61. 10.1152/japplphysiol.01234.2003 10.1152/japplphysiol.01307.2003 15247162

[45] Del Favero S, Roschel H, Artioli G, Ugrinowitsch C, Tricoli V, Costa A, Barroso R, Negrelli AL, Otaduy MC, Da Costa Leite C, Lancha-Junior AH, Gualano B. Creatine but not betaine supplementation increases muscle phosphorylcreatine content and strength performance. Amino Acids. 2012;42:2299–305. 10.1007/s00726-011-0972-5 10.1007/s00726-011-0972-5 21744011

[46] Candow DG, Vogt E, Johannsmeyer S, Forbes SC, Farthing JP. Strategic creatine supplementation and resistance training in healthy older adults. Appl Physiol Nutr Metab. 2015;40:689–94. 10.1139/apnm-2014-0498 10.1139/apnm-2014-0498 25993883

[47] Branch JD. Effect of creatine supplementation on body composition and performance: a meta-analysis. Int J Sport Nutr Exerc Metab. 2003;13:198–226. 10.1123/ijsnem.13.2.198 10.1123/ijsnem.13.2.198 12945830

[48] Aagaard P, Suetta C, Caserotti P, Magnusson SP, Kjær M. Role of the nervous system in sarcopenia and muscle atrophy with aging: strength training as a countermeasure. Scand J Med Sci Sports. 2010;20:49–64. 10.1111/j.1600-0838.2009.01084.x 10.1111/j.1600-0838.2009.01084.x 20487503

[49] Piasecki M, Ireland A, Jones DA, McPhee JS. Age-dependent motor unit remodelling in human limb muscles. Biogerontology. 2016;17:485–96. 10.1007/s10522-015-9627-3 10.1007/s10522-015-9627-3 PMC4889636 26667009

[50] Landi F, Calvani R, Cesari M, Tosato M, Martone AM, Ortolani E, et al. Sarcopenia: an overview on current definitions, diagnosis and treatment. Curr Protein Pept Sci. 2017;18: [Epub ahead of print]. 10.2174/1389203718666170607113459 10.2174/1389203718666170607113459 28595526

