# Supplementary material for: Combined creatine and HMB co-supplementation improves functional strength independent of muscle mass in physically active older adults: a randomized crossover trial
Source: GeroScience. 2025 Oct 10;48(3):4457–75. doi: 10.1007/s11357-025-01889-y (PMC13356226; doi:10.1007/s11357-025-01889-y)
Supplement: Supplementary file 1 — (DOCX 47.6 KB) [file 11357_2025_1889_MOESM1_ESM.docx]

| Supplementary Table S1. Detailed description of the nine multicomponent circuit (MCC) training sessions performed during the intervention. | | | |
| --- | --- | --- | --- |
| Station | **Exercise** | **Capacity** | **Work/Rest** |
| MCC1 | | | |
| 1 | Jump squats | Lower-body strength + power | 40´´/ 20´´ |
| 2 | High-knee run (on spot) | Cardio + speed | 40´´/ 20´´ |
| 3 | Bench press (barbell/dumbbells) | Upper-body strength | 10–12 reps / 20´´ |
| 4 | Balance walk on bench/low bar | Dynamic balance | 30–40´´ / 20´´ |
| 5 | Barbell deadlift | Global strength + core | 8–10 reps / 20´´ |
| 6 | Agility ladder (lateral/diagonal) | Coordination + speed | 40´´ / 20´´ |
| 7 | Pull-ups / Lat pulldown | Back strength + coordination | 8–10 reps / 20´´ |
| 8 | Burpees | Cardio + global strength | 30–40´´ / 20´´ |
| 9 | Dynamic stretches (lunges + torso rotation) | Flexibility + mobility | 30–40´´ / 20´´ |
| 10 | Lateral plank walk | Core + stability | 30–40´´ / 20´´ |
| 11 | Wall ball throws | Power + coordination | 12–15 reps / 20´´ |
| 12 | Lateral miniband walk + sprint | Glute strength + agility | 30–40´´ / 20´´ |
| MCC2 | | | |
| 1 | Jump lunges | Lower-body strength + power | 30–40´´/ 20´´ |
| 2 | Barbell/Dumbbell row | Back strength + core | 8–12 reps / 20´´ |
| 3 | Jump rope / double unders | Cardio + coordination | 40´´ / 20´´ |
| 4 | Shoulder-tap plank | Core + stability | 30–40´´ / 20´´ |
| 5 | Clap push-ups | Upper-body strength + power | 10–12 reps / 20´´ |
| 6 | Skater jumps | Coordination + lower-body power | 30–40´´ / 20´´ |
| 7 | Biceps curl (barbell/dumbbells) | Analytical arm strength | 10–12 reps / 20´´ |
| 8 | Mountain climbers | Cardio + core | 30–40´´/ 20´´ |
| 9 | Single-leg Romanian deadlift | Strength + balance | 8–10 reps / 20´´ |
| 10 | Battle ropes | Cardio + arms/core strength | 30´´ / 20´´ |
| 11 | Military press (dumbbells) | Shoulder strength + core | 8–12 reps / 20´´ |
| 12 | Russian twists (medicine ball) | Core rotation | 30–40´´ / 20´´ |
| MCC 3 | | | |
| 1 | Jump lunges | Lower-body strength + power | 30–40´´ / 20´´ |
| 2 | Battle ropes | Cardio + arm/core strength | 30´´ / 20´´ |
| 3 | Military press (dumbbells) | Shoulder strength + core | 10–12 reps / 20´´ |
| 4 | Lateral jumps over step | Coordination + agility | 30–40´´ / 20´´ |
| 5 | Kettlebell swings | Global strength + power | 12–15 reps / 20´´ |
| 6 | Zig-zag cone runs | Coordination + speed | 30–40´´ / 20´´ |
| 7 | Triceps dips (bench) | Arm strength | 10–12 reps / 20´´ |
| 8 | Mountain climbers | Cardio + core | 30–40´´ / 20´´ |
| 9 | Posterior chain mobility (inch walk, good mornings) | Flexibility + mobility | 30–40´´ / 20´´ |
| 10 | Shoulder-tap plank | Core + coordination | 30–40´´/ 20´´ |
| MCC4 | | | |
| 1 | Front squat (barbell/dumbbells) | Lower-body strength | 8–12 reps / 20´´ |
| 2 | Short sprints (20 m) | Cardio + speed | 30´´ / 20´´ |
| 3 | Incline press (dumbbells) | Chest + shoulder strength | 8–12 reps / 20´´ |
| 4 | Skater jumps | Coordination + lower-body power | 30–40´´ / 20´´ |
| 5 | Barbell deadlift | Posterior-chain strength | 8–10 reps / 20´´ |
| 6 | Agility ladder drills | Coordination + speed | 30–40´´ / 20´´ |
| 7 | Biceps curl (barbell/dumbbells) | Analytical arm strength | 10–12 reps / 20´´ |
| 8 | Burpees | Cardio + global strength | 30–40´´ / 20´´ |
| 9 | Dynamic lunges + torso rotation | Flexibility + mobility | 30–40´´ / 20´´ |
| 10 | Russian twists (medicine ball) | Core rotation | 30–40´´ / 20´´ |
| MCC 5 | | | |
| 1 | Bulgarian split squat (dumbbells) | Lower-body strength | 8–12 reps / 20´´ |
| 2 | Sled push/sprint | Cardio + global strength | 20–30 m / 30´´ |
| 3 | Arnold press (dumbbells) | Shoulder + chest strength | 8–12 reps / 20´´ |
| 4 | Box jumps | Lower-body power + agility | 8–10 reps / 20´´ |
| 5 | Inverted row (bar low) | Back + arm strength | 8–12 reps / 20´´ |
| 6 | Assault bike / Row ergometer | Cardio endurance | 30–40´´ / 30´´ |
| MCC 6 | | | |
| 1 | Squat (barbell/dumbbells) | Lower-body strength | 8–12 reps / 20´´ |
| 2 | Step-ups with jump | Lower-body power | 30–40´´/ 20´´ |
| 3 | Mountain climbers | Core + cardio | 30–40´´ / 20´´ |
| 4 | Romanian deadlift | Posterior-chain strength | 8–12 reps / 20´´ |
| 5 | High knees | Cardio + speed | 30´´ / 20´´ |
| 6 | Front plank | Core + stability | 30–40´´ / 20´´ |
| MCC 7 | | | |
| 1 | Bench press | Chest + triceps strength | 8–12 reps / 20´´ |
| 2 | Pull-ups / Lat pulldown | Back + biceps strength | 8–10 reps / 20´´ |
| 3 | Jump rope | Cardio endurance | 40´´ / 20´´ |
| 4 | Triceps dips (bench) | Arm strength | 10–12 reps / 20´´ |
| 5 | Row (barbell/dumbbells) | Back strength | 8–12 reps / 20´´ |
| 6 | Side plank | Core + obliques | 30–40´´ / 20´´ |
| MCC 8 | | | |
| 1 | Alternating lunges | Lower-body strength | 8–12 reps / 20´´ |
| 2 | Battle ropes | Cardio + arms/core | 30–40´´ / 20´´ |
| 3 | Military press (dumbbells) | Shoulder strength + core | 8–12 reps / 20´´ |
| 4 | Burpees | Global strength + cardio | 30–40´´ / 20´´ |
| 5 | Deadlift + row combo | Posterior-chain global strength | 8–10 reps / 20´´ |
| 6 | Russian twists | Core rotation | 30–40´´ / 20´´ |
| MCC 9 | | | |
| 1 | Hip thrust (barbell) | Glute strength | 10–12 reps / 20´´ |
| 2 | Clap push-ups | Chest + arm power | 10–12 reps / 20´´ |
| 3 | Short sprint (10–15 m) | Cardio + speed | 30´´ / 20´´ |
| 4 | Biceps curl | Arm strength | 10–12 reps / 20´´ |
| 5 | Lateral raises | Shoulder strength | 10–12 reps / 20´´ |
| 6 | Ab wheel rollout | Core deep strength | 8–12 reps / 20´´ |
| reps: Repetitions | | | |

| **Supplementary Table 2**. Baseline (T1, before exposure to any intervention) body composition values for the total sample. males. and females. | | | | | | | | | |
| --- | --- | --- | --- | --- | --- | --- | --- | --- | --- |
|  | **Total Sample (n=30)** | | | **Male (n=20)** | | | **Female (n=10)** | | |
| **Variable** | **CRE+HMB (n = 15)** | **Placebo (n = 15)** | **P** | **CRE+HMB (n = 10)** | **Placebo (n = 10)** | **P** | **CRE+HMB (n = 5)** | **Placebo (n = 5)** | **P** |
| Weight (kg) | 75.09 ± 15.05 | 78.97 ± 11.87 | 0.468 | 82.44 ± 12.90 | 82.89 ± 9.73 | 0.962 | 61.62 ± 7.46 | 69.18 ± 12.15 | 0.604 |
| BMI (kg/m²) | 26.58 ± 4.58 | 26.39 ± 3.73 | 0.862 | 28.32 ± 4.54 | 26.48 ± 2.26 | 0.989 | 23.38 ± 2.63 | 26.18 ± 6.70 | 0.257 |
| Fat Mass (kg) | 16.44 ± 7.93 | 17.87 ± 6.75 | 0.637 | 17.01 ± 9.27 | 16.70 ± 6.10 | 0.950 | 15.40 ± 5.22 | 20.80 ± 8.37 | 0.644 |
| Fat-Free Mass (kg) | 58.64 ± 11.04 | 61.04 ± 9.97 | 0.550 | 65.43 ± 6.52 | 66.11 ± 5.92 | 0.883 | 46.20 ± 4.25 | 48.38 ± 5.12 | 0.682 |
| Total Muscle Mass (kg) | 55.70 ± 10.52 | 58.01 ± 9.50 | 0.546 | 62.16 ± 6.21 | 62.85 ± 5.63 | 0.872 | 43.85 ± 4.05 | 45.93 ± 4.86 | 0.678 |
| Skeletal Muscle Mass (kg) | 33.19 ± 6.25 | 34.58 ± 5.64 | 0.543 | 37.03 ± 3.70 | 37.45 ± 3.34 | 0.865 | 26.17 ± 2.41 | 27.40 ± 2.90 | 0.684 |
| Appendicular Skeletal Muscle Mass (kg) | 24.46 ± 5.94 | 26.01 ± 5.59 | 0.478 | 28.01 ± 3.98 | 28.92 ± 3.30 | 0.640 | 17.97 ± 1.66 | 18.75 ± 2.08 | 0.761 |
| Muscle Mass Index (kg/m²) | 15.41 ± 3.39 | 14.43 ± 3.84 | 0.485 | 15.82 ± 3.57 | 13.80 ± 2.53 | 0.102 | 14.67 ± 3.20 | 16.00 ± 6.32 | 0.927 |
| Skeletal Muscle Index (kg/m²) | 8.60 ± 1.69 | 8.63 ± 1.36 | 0.974 | 9.57 ± 1.18 | 9.24 ± 0.67 | 0.351 | 6.82 ± 0.64 | 7.10 ± 1.50 | 0.773 |
| ALM/BMI | 0.92 ± 0.17 | 0.99 ± 0.21 | 0.281 | 1.00 ± 0.15 | 1.10 ± 0.13 | 0.152 | 0.77 ± 0.08 | 0.74 ± 0.12 | 0.387 |
| All values are expressed as mean ± standard deviation.  P: Comparisons were performed using univariate analysis with the intervention group as a fixed factor and age as a covariate. | | | | | | | | | |

| **Supplementary Table 3.** Baseline (T1) functional strength values for the total sample. males. and females. | | | | | | | | | |
| --- | --- | --- | --- | --- | --- | --- | --- | --- | --- |
|  | **Total Sample (n=30)** | | | **Male (n=20)** | | | **Female (n=10)** | | |
| **Variable** | **CRE+HMB (n = 15)** | **Placebo (n = 15)** | **P** | **CRE+HMB (n = 10)** | **Placebo (n = 10)** | **P** | **CRE+HMB (n = 5)** | **Placebo (n = 5)** | **P** |
| Grip Strength (kg) | 32.99 ± 9.04 | 33.62 ± 8.68 | 0.792 | 38.23 ± 6.33 | 37.46 ± 6.98 | 0.560 | 23.38 ± 3.20 | 24.03 ± 2.87 | 0.533 |
| Leg and Back Dyna Strength (kg) | 76.63 ± 19.87 | 79.71 ± 21.67 | 0.663 | 84.81 ± 18.37 | 88.89 ± 18.17 | 0.678 | 61.64 ± 13.11 | 56.79 ± 7.93 | 0.827 |
| Arms Flexion Dyna Strength (kg) | 33.60 ± 12.38 | 40.51 ± 13.22 | 0.133 | 39.30 ± 11.54 | 46.13 ± 11.20 | 0.167 | 23.15 ± 4.63 | 26.45 ± 3.46 | 0.205 |
| Dumbbell Arm Flexion (30 sec) | 26.29 ± 5.49 | 25.50 ± 6.14 | 0.713 | 27.73 ± 6.59 | 24.40 ± 4.22 | 0.152 | 29.33 ± 2.73 | 21.25 ± 10.01 | 0.305 |
| Crunches (30 sec) | 24.59 ± 5.76 | 22.07 ± 7.04 | 0.313 | 24.18 ± 6.01 | 21.80 ± 6.43 | 0.365 | 25.33 ± 5.72 | 22.75 ± 9.50 | 0.696 |
| Push-up (30 sec) | 15.59 ± 5.88 | 12.29 ± 5.51 | 0.133 | 16.91 ± 5.66 | 13.50 ± 5.21 | 0.112 | 13.17 ± 5.98 | 9.25 ± 5.74 | 0.696 |
| Isometric Pull-ups (sec) | 14.41 ± 11.59 | 11.20 ± 8.29 | 0.390 | 16.43 ± 12.44 | 14.80 ± 8.41 | 0.677 | 11.88 ± 7.97 | 1.71 ± 2.55 | 0.144 |
| All values are expressed as mean ± standard deviation.  P: Comparisons were performed using univariate analysis with the intervention group as a fixed factor and age as a covariate. | | | | | | | | | |
